# Pediatric thrombosis: Risk factors, diagnosis, and prevention strategies

**DOI:** 10.1097/MD.0000000000043370

**Published:** 2025-07-18

**Authors:** Emmanuel Ifeanyi Obeagu

**Affiliations:** a Department of Biomedical Laboratory Science, Africa University, Mutare, Zimbabwe.

**Keywords:** anticoagulation, diagnosis, pediatric thrombosis, prevention, risk factors

## Abstract

Pediatric thrombosis is an underdiagnosed condition with potentially life-threatening consequences. Unlike adult thrombosis, the pediatric variant is characterized by age-specific risk factors, such as central venous catheters, infections, and genetic predispositions, which demand a tailored approach to diagnosis and management. Neonates and adolescents are particularly vulnerable due to physiological and hormonal changes, respectively, that amplify their susceptibility to thrombotic events. A deeper understanding of these factors is crucial for early detection and effective prevention strategies. The diagnostic process for pediatric thrombosis is fraught with challenges owing to the nonspecific nature of symptoms and variability in presentation across age groups. Imaging modalities, such as Doppler ultrasound and computed tomography pulmonary angiography, remain the cornerstone for identifying thrombotic events. However, laboratory investigations, including thrombophilia testing and d-dimer assays, are essential adjuncts, particularly in cases of idiopathic or recurrent thrombosis. Timely and accurate diagnosis is pivotal for reducing morbidity and mortality associated with this condition.

## 1. Introduction

Pediatric thrombosis, although less common than its occurrence in adults, represents a significant clinical challenge due to its potential for severe morbidity and mortality. Thrombotic events in children can lead to complications such as pulmonary embolism (PE), stroke, and long-term vascular damage, underscoring the importance of early recognition and effective management. The epidemiology of pediatric thrombosis has been increasingly understood in recent years, with estimates suggesting an incidence of 0.07 to 0.49 cases per 10,000 children annually, though this varies across age groups and clinical settings. This condition’s low overall prevalence often results in delayed diagnosis and suboptimal treatment, making it a critical area of study.^[1,2]^ The etiopathogenesis of pediatric thrombosis differs markedly from that of adults, driven by a complex interplay of developmental, genetic, and environmental factors. Unlike adults, where thrombosis is predominantly associated with atherosclerosis and hypercoagulable states, pediatric cases are often linked to secondary triggers. These triggers include hospitalization, the presence of CVCs, malignancy, and congenital or acquired thrombophilia. The age-dependent variability in coagulation system maturity further complicates risk stratification and therapeutic interventions in children.^[3]^ CVCs remain the most prominent risk factor for thrombosis in pediatric patients, especially neonates and critically ill children. Up to 90% of thrombotic events in hospitalized pediatric populations are associated with CVCs, highlighting the necessity of effective catheter management protocols. Beyond CVCs, prolonged immobilization, surgical interventions, and infections contribute to a heightened thrombotic risk. The unique hemostatic system of neonates, characterized by reduced levels of natural anticoagulants and fibrinolytic activity, further predisposes this age group to thrombosis.^[4]^

In addition to acquired risk factors, genetic predispositions play a substantial role in pediatric thrombosis. Thrombophilic conditions such as Factor V Leiden mutation, prothrombin G20210A mutation, and deficiencies in natural anticoagulants like antithrombin, protein C, and protein S are significant contributors. A family history of thrombosis serves as a red flag, necessitating thorough genetic evaluation in affected children.^[5]^ While the presence of these inherited risk factors may not invariably result in thrombosis, they amplify the likelihood when combined with acquired triggers, forming the basis of a “2-hit hypothesis.” The clinical presentation of pediatric thrombosis varies significantly depending on the site and extent of the thrombus. For example, deep vein thrombosis (DVT) often manifests with swelling, erythema, and pain in the affected limb, while PE may present with respiratory distress and hypoxia. Neonates and younger children frequently exhibit nonspecific symptoms, complicating timely diagnosis. These variations necessitate a high degree of clinical suspicion and tailored diagnostic approaches to ensure early detection and intervention.^[6]^ Advancements in diagnostic imaging and laboratory techniques have significantly improved the ability to detect thrombosis in pediatric populations. Doppler ultrasound is widely regarded as the gold standard for evaluating venous thrombosis in extremities, while computed tomography (CT) pulmonary angiography is preferred for detecting PE. Magnetic resonance imaging (MRI) plays a critical role in diagnosing cerebral venous sinus thrombosis, particularly in neonates. Alongside imaging, laboratory tests such as d-dimer assays and thrombophilia panels are invaluable for evaluating the underlying risk factors and confirming the diagnosis.^[5]^ Prevention remains a cornerstone in managing pediatric thrombosis, focusing on mitigating modifiable risk factors and implementing prophylactic measures. Strategies such as regular assessment and prompt removal of CVCs, use of anticoagulant prophylaxis in high-risk settings, and promoting mobility in hospitalized children are critical interventions. Education and training for healthcare providers and families are equally important in fostering early recognition of symptoms and prompt medical attention.^[6]^

### 1.1. Aim

The aim of this review is to provide a comprehensive overview of pediatric thrombosis, focusing on its risk factors, diagnostic challenges, and effective prevention strategies.

### 1.2. Rationale

Pediatric thrombosis, though less common than in adults, is a significant and often under-recognized condition that can lead to severe long-term consequences if not properly managed. The pathophysiology of thrombosis in children differs from that in adults, with unique risk factors, including congenital conditions, malignancies, and the effects of treatments like chemotherapy and surgery. The rarity and complexity of pediatric thrombosis make it essential to have specialized, evidence-based management approaches tailored to this vulnerable population.^[7,8]^ Despite growing awareness, challenges remain in the timely diagnosis, treatment, and prevention of thrombosis in children. The limited availability of pediatric-specific data on anticoagulants, risk assessment tools, and treatment regimens further complicates clinical decision-making. Additionally, pediatric thrombosis often requires long-term management, which presents additional risks, such as bleeding complications, that must be carefully balanced with the need to prevent further thrombotic events.^[9]^ Given these challenges, there is a critical need for up-to-date, comprehensive guidelines that consider the unique aspects of pediatric thrombosis. A deeper understanding of the condition, including the identification of novel biomarkers, optimal therapeutic strategies, and the effectiveness of thromboprophylaxis in high-risk populations, will enhance clinical outcomes. This review seeks to fill the gap in existing literature by consolidating current knowledge, offering practical recommendations for management, and encouraging continued research to improve care for children affected by thrombosis. Ultimately, this review will contribute to raising awareness, optimizing treatment strategies, and improving the prognosis of pediatric patients with thrombosis, with the aim of reducing morbidity and enhancing their quality of life.

## 2. Materials and methods

The methodology employed in this review was designed to provide a thorough and comprehensive synthesis of existing literature on pediatric thrombosis, focusing on its risk factors, diagnostic challenges, prevention strategies, and management considerations. The approach consisted of several systematic steps to ensure that the most relevant, recent, and high-quality evidence was included. Below is a detailed description of the methodology used in this review. Literature Search Strategy A structured and comprehensive search of the literature was conducted to identify relevant studies, reviews, and clinical guidelines on pediatric thrombosis. The search was performed across multiple electronic databases, including PubMed, Scopus, and Google Scholar. The following key terms were used in various combinations: “pediatric thrombosis,” “childhood thrombosis,” “risk factors,” “diagnosis,” “prevention strategies,” “management,” “treatment,” and “thrombophilia in children.” Studies were selected based on their relevance to the topic, quality of the research, and contribution to understanding the diagnosis and management of pediatric thrombosis. Both primary research articles and review articles were included to provide a broad perspective on the subject.

### 2.1. Inclusion and exclusion criteria

- **Inclusion criteria**:
  - Studies focusing on pediatric populations (neonates, infants, children, and adolescents).
  - Research addressing any aspect of pediatric thrombosis, including risk factors, pathophysiology, diagnostic methods, prevention strategies, and treatment options.
  - Studies published in peer-reviewed journals.
  - Articles written in English.
- **Exclusion criteria**:
  - Studies focusing solely on adult populations.
  - Non-peer-reviewed articles or opinion pieces.
  - Articles not directly related to pediatric thrombosis or its management.

### 2.2. Critical appraisal

Each article included in the review was critically appraised for its methodological quality and relevance. Studies were evaluated based on their design, sample size, statistical analysis, and the robustness of their findings. This step ensured that only high-quality evidence was included in the final synthesis, providing a reliable foundation for the review’s conclusions. In addition, attention was paid to the generalizability of the study populations and the applicability of findings to clinical practice, particularly in diverse pediatric settings. The limitations and strengths of each study were also considered when drawing conclusions.

### 2.3. Ethical approval

No ethical approval was obtained as this is a narrative review.

## 3. Risk factors

Pediatric thrombosis arises from a complex interaction of acquired, genetic, and age-specific risk factors. These factors often overlap, creating a multifaceted etiology that necessitates careful evaluation and tailored interventions.

### 3.1. Acquired risk factors

Acquired conditions are among the most common contributors to pediatric thrombosis, with CVCs being the most prominent. The use of CVCs in critically ill neonates and children is a necessary intervention for administering medication and fluids, yet it significantly increases the risk of thrombus formation. Prolonged hospitalization, immobilization, surgical procedures, and mechanical ventilation also contribute to thrombotic risk by disrupting normal blood flow and enhancing a procoagulant state.^[10,11]^ Inflammatory conditions, including sepsis, Kawasaki disease, and systemic lupus erythematosus (SLE), are associated with increased thrombosis risk due to endothelial damage and hypercoagulability. Medications such as asparaginase, commonly used in leukemia treatment, and oral contraceptives in adolescents further amplify this risk. Additionally, obesity and sedentary behavior, especially during adolescence, are emerging risk factors that warrant attention in the prevention of thrombotic events.^[12]^

### 3.2. Genetic predispositions

Inherited thrombophilia plays a significant role in the etiology of pediatric thrombosis, especially in cases of recurrent or idiopathic thrombotic events. Common genetic predispositions include Factor V Leiden mutation, prothrombin G20210A mutation, and deficiencies in natural anticoagulants such as antithrombin, protein C, and protein S. Children with these conditions may remain asymptomatic until exposed to an additional acquired risk factor, highlighting the importance of the “2-hit hypothesis” in understanding pediatric thrombosis. Family history of thrombosis is another critical genetic marker. A positive family history often prompts genetic testing and risk stratification in children presenting with thrombotic events. While not all genetic mutations guarantee the development of thrombosis, their presence significantly increases vulnerability, necessitating close monitoring and, in some cases, prophylactic measures.^[13,14]^

### 3.3. Age-specific factors

Age plays a pivotal role in determining the risk profile for thrombosis in pediatric populations. In neonates, the hemostatic system is developmentally distinct, characterized by lower levels of coagulation factors and natural anticoagulants. These physiological differences predispose neonates to thrombosis, particularly in the presence of risk factors such as prematurity, sepsis, or the use of umbilical catheters. In contrast, adolescents face a unique set of risks driven by hormonal changes, lifestyle factors, and exposure to certain medications. Puberty-associated hormonal shifts can promote a prothrombotic state, particularly in females using oral contraceptives. Lifestyle-related factors, such as obesity and prolonged immobility due to screen time, also contribute to an increased risk of thrombosis in this age group.^[15,16]^

### 3.4. Multifactorial considerations

Many cases of pediatric thrombosis are multifactorial, with both acquired and genetic factors contributing to the development of the condition. For example, a child with a genetic predisposition to thrombophilia who undergoes surgery or prolonged hospitalization is at significantly higher risk. This interplay underscores the need for comprehensive risk assessments that incorporate both clinical history and laboratory findings to guide preventive and therapeutic interventions.^[17]^

## 4. Diagnosis

Diagnosing pediatric thrombosis presents unique challenges due to the variable clinical presentation, age-specific physiological differences, and the rarity of the condition compared to adult populations. Timely and accurate diagnosis is essential to initiate appropriate treatment and prevent complications, including long-term morbidity. The diagnostic process integrates clinical evaluation, imaging modalities, and laboratory investigations to confirm the presence of thrombosis and determine its underlying cause.

### 4.1. Clinical presentation

The clinical manifestations of thrombosis in children vary based on the site and severity of the thrombus. DVT often presents with localized swelling, pain, and erythema in the affected limb. PE, a potentially life-threatening complication, may present with respiratory distress, chest pain, hypoxia, and, in severe cases, hemodynamic instability. In neonates, the symptoms are frequently nonspecific, such as fussiness, feeding difficulties, or unexplained desaturation, complicating early recognition. In older children, thrombosis associated with CVCs may present as catheter dysfunction or edema in the area drained by the affected vein. The variability in symptoms necessitates a high index of suspicion, particularly in high-risk populations such as hospitalized or critically ill children.^[18,19]^

### 4.2. Imaging modalities

Imaging studies are critical for confirming the presence and extent of thrombosis. The choice of imaging modality depends on the suspected site of thrombosis, the child’s age, and clinical considerations such as radiation exposure:

- **Ultrasound with Doppler:** This is the first-line imaging technique for evaluating DVT. It is noninvasive, widely available, and highly sensitive for detecting thrombi in extremity veins.^[20]^
- **CT pulmonary angiography:** Considered the gold standard for diagnosing PE, this technique offers excellent sensitivity and specificity but involves ionizing radiation, which is a concern in pediatric populations.^[21]^
- **MRI:** Preferred for conditions such as cerebral venous sinus thrombosis due to its superior visualization of intracranial structures without radiation. MRI may also be used for venous thrombosis in other deep or atypical sites.^[22]^
- **Venography:** Although rarely used due to its invasive nature, venography remains an option for complex cases where noninvasive imaging is inconclusive.^[23,24]^

### 4.3. Laboratory investigations

Laboratory tests play a complementary role in diagnosing thrombosis and identifying underlying risk factors:

- **d****-dimer:** Elevated d-dimer levels suggest the presence of active thrombus formation and breakdown. However, the test lacks specificity in children due to variations in baseline levels and the influence of comorbidities, such as infections.^[25]^
- **Thrombophilia testing:** This includes evaluating for inherited conditions such as Factor V Leiden mutation, prothrombin gene mutation, and deficiencies in natural anticoagulants (antithrombin, protein C, and protein S). Testing is particularly important in cases of recurrent thrombosis or when a family history of thrombophilia is present.^[26]^
- **Inflammatory markers:** Elevated levels of C-reactive protein or erythrocyte sedimentation rate may indicate an inflammatory or infectious trigger for thrombosis.^[27]^

### 4.4. Diagnostic challenges

The diagnosis of pediatric thrombosis is often delayed due to its nonspecific presentation and the rarity of the condition. Misdiagnosis or underdiagnosis is common, particularly in neonates and young children, who may not exhibit classic symptoms. Additionally, the technical limitations of imaging in small children, coupled with the lack of standardized age-specific reference ranges for laboratory markers, add to the complexity.

### 4.5. Multidisciplinary approach

Accurate diagnosis often requires collaboration among pediatricians, hematologists, radiologists, and other specialists. A thorough clinical history, including a review of potential risk factors such as recent surgeries, infections, or family history of thrombosis, is crucial. Tailored diagnostic protocols based on age, clinical context, and available resources can enhance early detection and improve outcomes.

## 5. Children affected by perinatal arterial ischemic stroke

Perinatal arterial ischemic stroke (PAIS) is a devastating cerebrovascular event that occurs between the 20th week of gestation and the 28th postnatal day. Though relatively rare, its impact on infants and their families is profound and enduring. Unlike stroke in older populations, PAIS occurs at a critical time when the neonatal brain is still in the early stages of development, rendering it vulnerable to permanent injury. Most often, PAIS affects term infants and involves large cerebral arteries – most notably the middle cerebral artery.^[28]^ The presentation of PAIS is often subtle, delayed, or misattributed. In the first hours to days after birth, the neonate may appear clinically well. Then, unexpectedly, a focal seizure may occur – often on the second or third day of life – becoming the earliest indication of cerebral insult. These seizures, frequently unilateral, serve as a red flag and often prompt neuroimaging, which reveals the stroke. MRI remains the gold standard in identifying the affected vascular territory and estimating the extent of brain injury.^[29]^ The causes of PAIS are multifactorial. Maternal, placental, and neonatal factors intertwine to create a prothrombotic milieu. Maternal preeclampsia, chorioamnionitis, thrombophilia, prolonged rupture of membranes, and placental abnormalities have all been associated. Neonatal conditions such as congenital heart disease, infections, and inherited clotting disorders also contribute to the risk. In many cases, however, no single definitive cause is identified, complicating prevention efforts.^[30]^

For the affected child, PAIS is not a transient event – it sets the stage for lifelong challenges. Hemiparetic cerebral palsy is a common long-term outcome, especially when the stroke occurs in the territory of the left middle cerebral artery. In addition to motor deficits, cognitive impairments, language delays, and behavioral disorders may emerge as the child grows. The full spectrum of impact often unfolds over years, requiring continuous monitoring and early intervention.^[31]^ Rehabilitation begins early. Neonatal care teams work closely with neurologists, physiotherapists, and occupational therapists to support neurodevelopment. Early interventions, including constraint-induced movement therapy and motor training, have shown promise in improving functional outcomes. Despite these efforts, many families face an uphill journey, grappling with uncertainty about their child’s future, financial burdens, and the emotional toll of raising a child with special needs.^[32]^ Importantly, the story of children with PAIS is not just one of disability – it is also one of resilience. With advances in neonatal care, early diagnosis, and comprehensive therapy, many children show remarkable adaptation and developmental gains. Neuroplasticity in the infant brain offers a powerful window of hope; with the right support, some children achieve near-normal function and thrive in educational and social environments.^[33]^

## 6. Family and gestational history

Behind every case of PAIS lies a story – one that often begins long before birth, woven quietly through family genetics and the journey of pregnancy. Understanding the tapestry of family and gestational history offers critical insight into the causes, risks, and future implications of this life-altering condition.^[34]^ In many families, the birth of a child is anticipated with joy and preparation. Yet, unknown to many, inherited risks may silently accompany the developing fetus. Thrombophilic disorders – conditions that predispose individuals to abnormal clotting – may run in families undetected, as their effects might not manifest until challenged by a physiological stressor such as pregnancy or delivery. Mothers who carry genetic mutations like Factor V Leiden or prothrombin gene mutation may never know their status until a neonatal event, such as PAIS, brings it painfully into focus. Sometimes, there may be a history of unexplained miscarriages, clotting events, or cardiovascular disease in the family, subtle clues that now take on deeper significance in hindsight.^[35]^ Gestational history further deepens the understanding of perinatal stroke. The placenta – often called the “forgotten organ” – serves as the vital link between mother and fetus, and its dysfunction can set the stage for thrombotic events. In pregnancies complicated by preeclampsia, gestational hypertension, or placental abruption, the risk of impaired oxygen delivery and clot formation increases. For some mothers, the road to delivery is complicated by infections, such as chorioamnionitis, or prolonged labor, each of which can create inflammatory or procoagulant states that raise the likelihood of vascular complications in the neonate.^[29,36]^

The mode of delivery also plays a role in the broader context. Instrumental deliveries, emergency cesarean sections, or difficult labor may result in hemodynamic instability or vascular injury, predisposing the neonate to stroke. Additionally, neonatal factors such as congenital heart disease or systemic hypotension immediately after birth may emerge only at delivery, even when gestation seemed uncomplicated.^[37,38]^ Yet, despite meticulous prenatal care, PAIS can still occur in pregnancies that appear healthy. This uncertainty can be devastating for families, who often grapple with feelings of guilt or confusion. The lack of a definitive cause in many cases emphasizes the complexity of stroke pathogenesis in newborns, where subtle interactions between maternal, fetal, and environmental factors collide at a vulnerable time.^[39,40]^ In counseling families, taking a thorough family and gestational history is not merely a formality – it is a window into possible explanations and a guide for future pregnancies. Testing for thrombophilia, evaluating placental pathology, and reviewing obstetric records provide not only clarity but also a foundation for prevention and reassurance.^[41]^

## 7. Risk factors of perinatal stroke

Perinatal stroke is a quiet thief – it arrives unannounced, often without obvious warning, and can forever change the trajectory of a newborn’s life. As physicians, researchers, and families search for answers, 1 question echoes: *Why did this happen?* To understand PAIS, we must step back and examine the risk factors – those hidden currents that, when they converge, give rise to this cerebral storm.^[28]^ The causes of perinatal stroke are rarely straightforward. Instead, they arise from a complex interplay of maternal, fetal, placental, and environmental factors. While no single risk factor can fully explain every case, patterns begin to emerge when viewed through the lens of population studies and clinical experience.^[29]^ Maternal risk factors are often the starting point. Pregnancy is a prothrombotic state by design, intended to prevent excessive bleeding during delivery. However, when this physiological balance tips, the risk of clot formation rises. Conditions like preeclampsia, gestational diabetes, infections such as chorioamnionitis, or autoimmune diseases such as lupus may create a maternal environment conducive to clot formation. A history of infertility treatments, prolonged labor, or emergency cesarean section can further contribute to maternal vascular stress.^[30]^ In some cases, a silent hereditary thread is at play. Thrombophilia, whether inherited or acquired, may pass from parent to child, increasing the likelihood of clot formation in the fetal circulation. Factor V Leiden, prothrombin gene mutations, and protein C or S deficiencies may remain dormant until the stresses of delivery or fetal development expose their presence.^[31]^

Then there is the placenta – an organ often overlooked once the baby is born but central to perinatal health. Placental pathology in cases of perinatal stroke frequently reveals vascular infarcts, thrombi, or fibrin deposition. A compromised placenta may lead to diminished oxygen and nutrient delivery, creating a hypoxic-ischemic environment in which a vulnerable fetal brain becomes prone to injury.^[31]^ Fetal and neonatal factors also contribute their share to the risk equation. Congenital heart defects, which can lead to turbulent blood flow or embolic events, are known contributors. Perinatal infections, neonatal sepsis, and hypoxic-ischemic encephalopathy can further exacerbate inflammation and coagulation cascades in the neonate. Some strokes occur during or shortly after birth due to birth trauma, instrumental delivery, or cardiorespiratory instability, all of which can compromise cerebral perfusion.^[32]^ Yet, perhaps the most sobering reality is that in nearly half of all cases, no clear risk factor is found. These cryptogenic strokes remind us that our current understanding, though expanding, is still incomplete. They challenge clinicians to look deeper – into genetic markers, immune interactions, and microvascular development – to find the missing pieces.^[33]^ Identifying risk factors is not about assigning blame. Rather, it is about illumination – shining a light on the subtle signals that may have gone unnoticed. It is about guiding future pregnancies, refining neonatal care, and, above all, offering answers to families who find themselves asking *why*. Each discovery of a risk factor – no matter how small – represents a step toward prevention, early intervention, and hope.^[34]^ In the quiet corridors of neonatal intensive care units, as clinicians piece together histories and review scans, the presence of risk factors offers a map. Not a perfect one, but one that leads from uncertainty toward understanding. And for the families walking this road with their child, even the smallest clarity can be a source of strength.^[35]^

### 7.1. Gender, clinical profiles and outcomes

Each child affected by PAIS carries a unique story – yet patterns emerge across cases, offering valuable insights into how this early brain injury unfolds. Among these, gender has surfaced not only as a demographic detail but as a subtle influencer in the presentation and outcomes of the condition. Coupled with rich clinical data and the evolving journey of affected children, a clearer picture begins to form – one of resilience, challenges, and hopeful possibilities.^[42]^ Statistical trends have hinted at a slight male predominance in perinatal stroke. Although the reasons remain under investigation, researchers speculate that sex-specific differences in brain development, immune responses, and coagulation profiles may underlie this discrepancy. Male infants may be more vulnerable to hypoxic and ischemic injuries, possibly due to slower cerebral maturation or hormonal influences during the neonatal period. However, these biological nuances are still being explored in ongoing studies.^[43]^ Beyond gender, clinical data collected at presentation often reveal a dramatic or subtle onset. Many affected newborns appear well at birth, passing the usual milestones of appearance, pulse, grimace, activity, and respiration scores and immediate postnatal adaptation. Then, within the first hours to days, something changes. Seizures – often focal and resistant to first-line anticonvulsants – may be the first visible sign, drawing attention to an otherwise hidden neurological insult. In some cases, hypotonia, altered consciousness, or feeding difficulties prompt further evaluation.^[28]^ Imaging – most commonly cranial ultrasound followed by MRI – confirms the diagnosis, revealing a unilateral arterial infarct, often within the middle cerebral artery territory. The infarct may involve cortical and subcortical regions, sometimes extending to the basal ganglia or internal capsule, depending on the timing and severity of the event. EEG studies often demonstrate focal slowing or epileptiform activity, correlating with clinical seizure patterns.^[44]^

As the months unfold, outcomes vary widely. Some children recover with minimal or no overt deficits, while others face a lifelong journey of managing hemiplegic cerebral palsy, epilepsy, or cognitive and language delays. Early intervention makes a significant difference – those enrolled in physiotherapy, occupational therapy, and speech programs early tend to fare better in both motor and cognitive domains.^[45]^ A striking feature in follow-up data is the plasticity of the infant brain. Many affected children show remarkable ability to reorganize neural functions, especially when interventions are timely. However, this potential is not limitless. Some children develop poststroke epilepsy, which can be difficult to control, or behavioral challenges that emerge during school years. Parents often describe a gradual realization that their child’s pace of development is different – not necessarily broken, but uniquely theirs.^[46]^ Interestingly, outcome studies suggest that while boys may be more frequently affected, girls may have slightly better long-term recovery profiles, possibly due to neuroprotective effects of estrogen or differences in neural plasticity. Yet these trends are not rules – they are patterns that continue to evolve with more research, more follow-up, and more understanding.^[47]^ The clinical journey of a child with perinatal stroke is not a fixed trajectory – it is a winding path shaped by early medical care, rehabilitation, family support, and the mysteries of individual neurodevelopment. Gender and initial clinical data help inform prognosis, but they do not dictate destiny. What matters most is the early recognition, sustained support, and a holistic approach that sees beyond the injury to the whole child and their potential.^[48,49]^ For many families, the journey begins in uncertainty – but through compassionate care and evidence-based follow-up, it often leads to strength, adaptation, and hope.^[50]^

## 8. Prevention strategies

Thrombosis in children, though less common than in adults, poses a serious threat that can lead to significant complications if not properly managed. Preventing thrombosis in pediatric patients requires a comprehensive approach that includes early identification of risk factors, individualized care plans, and a combination of pharmacological and non-pharmacological interventions.

### 8.1. Identifying at risk children

The first and most important step in preventing pediatric thrombosis is the identification of children who are at increased risk. Children with congenital conditions such as protein C, protein S, or antithrombin deficiencies, as well as those with inherited thrombophilias like Factor V Leiden mutation or prothrombin gene mutations, are naturally more predisposed to developing thrombotic events. Therefore, understanding a child’s genetic background is crucial in assessing their risk. Neonates and infants, especially those born prematurely, are also at higher risk for thrombosis. Premature birth is associated with immaturity in both the vascular system and the coagulation cascade, which can increase the likelihood of thrombus formation. Additionally, the use of invasive procedures such as umbilical catheters or mechanical ventilation in these infants contributes to the risk. Identifying these high-risk newborns early on allows for targeted prevention strategies that can reduce the likelihood of thrombotic complications.^[51]^

### 8.2. Pharmacological interventions

For children who are deemed high-risk due to their medical conditions, surgery, or other factors, pharmacological prophylaxis becomes a critical component of prevention. The use of anticoagulant medications, such as low-molecular-weight heparin (LMWH), has become common practice in preventing venous thromboembolism in pediatric patients, particularly in those undergoing major surgeries or requiring CVCs. LMWH helps prevent clot formation by inhibiting certain clotting factors, and it is preferred in children because of its predictable pharmacokinetics and ease of use. For children who require long-term anticoagulation therapy, such as those with congenital thrombophilia or certain chronic medical conditions, medications like warfarin or direct oral anticoagulants (DOACs) may be considered. While warfarin has been traditionally used, its use requires regular monitoring of the child’s international normalized ratio (INR) to ensure therapeutic levels. DOACs, though less commonly used in pediatric patients, offer a promising alternative with fewer monitoring requirements. However, the use of anticoagulants in children must be carefully managed, as there is a fine line between preventing thrombosis and increasing the risk of bleeding. In children with contraindications to anticoagulation, such as those with active bleeding or certain bleeding disorders, alternative measures must be considered. One option is the use of an inferior vena cava (IVC) filter, which can be placed to prevent embolic events. However, the use of IVC filters is typically reserved for children who are unable to receive anticoagulation therapy due to significant risk factors.^[3,52]^

### 8.3. Non-pharmacological measures

Pharmacological strategies are crucial, but non-pharmacological measures also play an important role in preventing pediatric thrombosis. These measures are particularly important in hospitalized children or those recovering from surgery, as they help reduce immobility and promote circulation. Early mobilization is one of the simplest yet most effective ways to prevent thrombosis. In hospitalized children, particularly those recovering from surgery or trauma, encouraging early movement – such as passive or active range-of-motion exercises – can help stimulate blood flow and prevent the formation of clots. For older children who are able to move around more independently, encouraging regular walking and physical activity is essential for maintaining healthy circulation. For children who are immobile or at very high-risk, compression stockings or devices may be used to reduce the likelihood of clot formation. Graduated compression stockings apply pressure to the legs, promoting venous return and reducing the chances of DVT. In children who are unable to wear compression stockings, mechanical devices that periodically inflate and deflate around the legs can be used to achieve similar results. Hydration is another key factor in preventing thrombosis. Dehydration can lead to blood becoming more viscous, which increases the risk of clot formation. Ensuring that children remain well-hydrated, especially those who are hospitalized or recovering from surgery, is an important step in thrombosis prevention.^[53,54]^

### 8.4. Managing underlying medical conditions

Thrombosis prevention in children also requires a proactive approach to managing underlying medical conditions that can increase the risk of clot formation. For example, children with cancer are at a significantly higher risk of developing thrombosis, particularly as a result of chemotherapy, central venous lines, and immobilization during treatment. Proactive management of cancer therapies, including the use of thromboprophylaxis during high-risk periods, can significantly reduce the likelihood of venous thromboembolism. Children with nephrotic syndrome, a condition that leads to excessive protein loss in the urine, are also at risk of thrombosis due to changes in their blood clotting mechanisms. Similarly, children with autoimmune diseases like SLE often experience a hypercoagulable state that can predispose them to thrombosis. In these cases, the prevention of thrombosis involves a combination of pharmacological interventions, such as anticoagulation therapy, along with close monitoring of disease activity and management of inflammation. Children with obesity are increasingly recognized as a high-risk group for thrombosis. Obesity is associated with a number of risk factors, including insulin resistance, low-grade inflammation, and increased blood clotting factors. Preventing thrombosis in obese children requires addressing the underlying condition through lifestyle modifications, such as promoting healthy eating habits, increasing physical activity, and managing associated comorbidities like hypertension or diabetes.^[55,56]^

### 8.5. Education and family involvement

Finally, education plays a central role in preventing pediatric thrombosis. Parents and caregivers should be educated on the signs and symptoms of thrombosis, such as swelling, pain, or redness in a limb, sudden shortness of breath, or chest pain, so they can seek timely medical attention if necessary. This is particularly important for children with underlying risk factors who may be more susceptible to thrombotic events. In cases where thrombophilia is present in the family, parents should be educated about the potential risks and preventive measures. For children with known genetic risk factors, such as those with a family history of thrombosis, it is essential to provide counseling on how to manage the risk during periods of surgery, immobility, or other high-risk situations.^[57]^

### 8.6. Management considerations

Thrombosis in pediatric patients, while less frequent than in adults, presents unique challenges that require specialized management strategies. Managing pediatric thrombosis involves a careful balance between preventing clot formation, minimizing the risk of bleeding, and addressing underlying conditions that may predispose a child to thrombotic events. This narrative explores the key management considerations for pediatric thrombosis, highlighting the importance of early detection, individualized care, and the integration of multidisciplinary approaches to ensure optimal patient outcomes.

#### 8.6.1. Early diagnosis

Effective management of pediatric thrombosis begins with early and accurate diagnosis. Symptoms of thrombosis in children, such as unexplained swelling, pain, or redness in a limb, may be subtle and difficult to detect, particularly in neonates and infants. As a result, healthcare providers must maintain a high index of suspicion, especially in children with known risk factors such as congenital thrombophilia, cancer, or those who have undergone major surgery or prolonged immobilization. Advances in imaging technology, particularly ultrasonography and CT scans, have significantly improved the ability to diagnose thrombosis in pediatric patients. Duplex ultrasonography remains the gold standard for diagnosing DVT in children, as it is noninvasive, widely available, and reliable. For suspected PE, a CT pulmonary angiography or ventilation/perfusion scan can be used. MRI may also be considered in certain cases, particularly for diagnosing cerebral venous thrombosis or other central nervous system-related thromboses. Given that pediatric thrombosis can occur in different vascular territories – such as the veins, arteries, or even the microcirculation – early detection is critical in ensuring that children receive the appropriate interventions promptly. Once a diagnosis is confirmed, the treatment plan can be tailored based on the location and extent of the thrombus, the child’s underlying risk factors, and any comorbid conditions.^[58,59]^

#### 8.6.2. Anticoagulation therapy

Anticoagulation therapy is the cornerstone of managing pediatric thrombosis. The goal is to prevent further thrombus formation and reduce the risk of embolization while minimizing the potential for bleeding complications. The choice of anticoagulant, the duration of therapy, and the monitoring of treatment require careful consideration, as pediatric patients often have different pharmacokinetics and sensitivities compared to adults. For children with DVT or PE, LMWH is commonly used as the first-line treatment. LMWH is preferred in pediatric patients due to its predictable pharmacokinetics, ease of administration, and lower risk of bleeding compared to unfractionated heparin. It is typically administered subcutaneously, and its dose is adjusted based on the child’s weight and renal function. Once the initial acute phase is managed with LMWH, the patient may transition to an oral anticoagulant, such as warfarin or, in some cases, DOACs, depending on the clinical situation.^[60,61]^ Warfarin, while effective, requires regular monitoring of the INR to ensure therapeutic anticoagulation. Achieving and maintaining the appropriate INR range in children can be challenging due to their variable response to the drug. DOACs, such as rivaroxaban and apixaban, offer an alternative with fewer monitoring requirements, but their use in pediatrics is still evolving, and more data are needed to establish dosing guidelines and safety profiles in children. The duration of anticoagulation therapy varies depending on the underlying cause of the thrombosis. In most cases, anticoagulation therapy is continued for at least 3 to 6 months. However, children with inherited thrombophilias or those who experience recurrent thrombotic events may require long-term anticoagulation. In these instances, the risks of bleeding must be carefully weighed against the benefits of preventing further thrombosis. Close monitoring is essential to ensure that anticoagulation therapy remains within a therapeutic range and to promptly identify any bleeding complications.^[62]^

#### 8.6.3. Consideration of bleeding risks

While anticoagulation therapy is essential in managing pediatric thrombosis, it also increases the risk of bleeding, which is a significant concern in this patient population. Children, especially younger ones, are at higher risk for bleeding complications due to their smaller blood volume, greater skin fragility, and more delicate vasculature. As such, the decision to start anticoagulant therapy must be made with careful attention to the child’s bleeding risk factors. For children with active bleeding, significant trauma, or a history of bleeding disorders such as hemophilia or von Willebrand disease, anticoagulation therapy may be contraindicated or used with extreme caution. In such cases, alternative strategies, such as the use of an IVC filter, may be considered to prevent PE without the need for systemic anticoagulation. However, IVC filters are not without risks, including filter-related complications, and their use should be reserved for situations where anticoagulation is not feasible. In managing children with thrombosis and underlying bleeding risk, careful consideration should be given to the timing and dosing of anticoagulant therapy. For example, in cases of surgery or trauma, it may be appropriate to withhold anticoagulants temporarily until the child has recovered sufficiently. Conversely, in the context of cancer or other prothrombotic conditions, prophylactic anticoagulation may be initiated early to prevent clot formation, even in the absence of overt symptoms.^[63,64]^

#### 8.6.4. Management of underlying conditions

The management of pediatric thrombosis is not limited to anticoagulation alone; it must also involve the treatment of any underlying conditions contributing to the thrombotic risk. For instance, children with cancer who develop thrombosis as a complication of their disease or its treatment may require chemotherapy adjustments or the use of prophylactic anticoagulation during high-risk periods. Multidisciplinary care involving oncologists, hematologists, and thrombosis specialists is essential to optimize both cancer treatment and thrombosis prevention. For children with congenital thrombophilias, family history plays a significant role in shaping the management approach. Genetic counseling is recommended to help families understand the risks and preventive strategies for thrombosis in siblings and other relatives. In some cases, long-term anticoagulation may be required during periods of high-risk, such as following surgery, immobilization, or trauma. In contrast, children with acquired thrombophilias, such as those with nephrotic syndrome or autoimmune diseases like SLE, may benefit from disease-modifying therapies that address the underlying inflammatory or immune-mediated processes contributing to thrombosis.^[65,66]^

#### 8.6.5. Psychological and family support

The management of pediatric thrombosis requires not only medical intervention but also psychological and family support. A child diagnosed with thrombosis may experience significant anxiety, particularly if long-term anticoagulation therapy is required or if the thrombotic event leads to hospitalization or a major medical procedure. Support from a pediatric hematologist, as well as counseling for both the child and their family, can help alleviate stress and provide emotional coping strategies. Parents and caregivers should be thoroughly educated about the signs and symptoms of thrombosis, as well as the importance of adhering to anticoagulation therapy and follow-up appointments. Given the complexity of managing pediatric thrombosis, clear communication and education are vital to ensuring that families are empowered to manage their child’s care at home and recognize when to seek medical help.^[67,68]^

## 9. Recommendations for the management and prevention of pediatric thrombosis

Pediatric thrombosis is a complex and multifactorial condition that requires a comprehensive approach to management and prevention. Based on the current understanding of risk factors, diagnostic strategies, treatment options, and the unique needs of pediatric patients, the following recommendations are proposed to enhance the care and outcomes of children at risk for thrombosis.

### 9.1. Early and routine screening for at risk populations

Pediatric patients with known risk factors such as congenital or acquired thrombophilia, malignancy, prolonged immobility, or recent surgery should be routinely screened for thrombosis. Regular screening is especially important for children with chronic conditions that increase thrombotic risk, such as nephrotic syndrome, SLE, or those undergoing cancer treatment. Consider using validated thrombosis risk assessment tools in high-risk pediatric populations, particularly in hospitalized or critically ill children, to identify those who may benefit from prophylactic anticoagulation.^[69]^

### 9.2. Personalized anticoagulation therapy

Given the variations in pharmacokinetics between children and adults, anticoagulation therapy should be tailored to the individual child’s weight, age, renal function, and comorbid conditions. Monitoring should be adjusted according to the specific anticoagulant used (e.g., INR for warfarin, anti-Xa levels for LMWH). LMWH remains the preferred choice for initial treatment of pediatric thrombosis, given its predictable dosing and safety profile. Consider transitioning to DOACs where applicable, with careful consideration of the child’s age and the need for ongoing clinical trials to establish pediatric dosing and safety guidelines for these agents. For children with inherited thrombophilia or recurrent thrombotic events, long-term anticoagulation may be necessary. This decision should be made collaboratively with hematologists and other specialists, taking into account the risks of bleeding and the child’s overall prognosis.^[70]^

### 9.3. Multidisciplinary team approach

Pediatric thrombosis should be managed by a multidisciplinary team that includes hematologists, cardiologists, oncologists, nephrologists, and other specialists depending on the underlying cause of the thrombosis. This ensures that both the thrombotic event and any underlying medical conditions are addressed concurrently. For children with cancer, ensure that oncologists and hematologists collaborate to provide an integrated approach to prevent and manage thrombosis, particularly during high-risk treatment phases such as chemotherapy, stem cell transplant, or surgery.^[71]^

### 9.4. Thrombosis prophylaxis in high-risk settings

Hospitalized children, particularly those undergoing major surgery, intensive care, or prolonged immobilization, should receive appropriate thromboprophylaxis, such as LMWH, unless contraindicated. For children with cancer, thromboprophylaxis is often indicated during periods of chemotherapy-induced neutropenia or other high-risk phases. After surgery, pediatric patients should be closely monitored for signs of thrombosis. In high-risk surgical populations, such as those undergoing orthopedic or neurosurgical procedures, postoperative anticoagulation or mechanical prophylaxis should be considered.^[72]^

### 9.5. Timely and accurate diagnosis

Ensure the availability and use of advanced imaging techniques like duplex ultrasonography for diagnosing DVT, CT or MRI for suspected PE or cerebral venous thrombosis, and V/Q scans for PE when needed. Timely imaging is critical to prevent progression of thrombosis and related complications. Health professionals should maintain a high level of vigilance in recognizing the signs and symptoms of thrombosis, particularly in neonates and infants who may not exhibit overt symptoms. Parents and caregivers should also be educated on the warning signs of thrombosis and when to seek immediate medical attention.^[73]^

### 9.6. Balancing the risk of bleeding

A thorough bleeding risk assessment should be performed before initiating anticoagulation therapy. This includes evaluating the child’s medical history for any bleeding disorders or active bleeding. For children at high bleeding risk, alternative interventions such as IVC filters should be considered. In pediatric patients, bleeding complications should be managed promptly with a multidisciplinary approach. This includes careful monitoring of anticoagulation therapy and adjusting the dosage when necessary, especially in cases of trauma or surgery.

### 9.7. Psychosocial support for children and families

Diagnosis and treatment of thrombosis can cause significant psychological stress for both children and their families. Routine psychological support should be offered to help families cope with the emotional impact of a chronic condition requiring long-term anticoagulation therapy. Families should receive comprehensive education about thrombosis prevention, signs of recurrence, and the importance of medication adherence. Children, depending on their age, should also be educated on lifestyle modifications that can reduce their risk of thrombosis, such as staying active and avoiding prolonged immobility.

### 9.8. Regular follow-up and monitoring

Pediatric patients on anticoagulants require frequent follow-up visits to monitor for adverse effects such as bleeding or recurrent thrombotic events. Laboratory tests to monitor anticoagulant therapy (e.g., INR, anti-Xa levels) should be conducted regularly, and clinical follow-up should be scheduled at least every 3 to 6 months, depending on the patient’s specific circumstances. Children with underlying conditions such as congenital thrombophilias should be monitored regularly by pediatric hematologists. Lifestyle modifications, including managing obesity and maintaining hydration, should also be discussed as part of the long-term management strategy.

### 9.9. Research and development of pediatric-specific guidelines

There is a need for ongoing research into pediatric thrombosis, particularly in relation to the use of novel anticoagulants (such as DOACs) in children. Well-designed clinical trials are necessary to determine optimal dosing, safety profiles, and long-term outcomes of these therapies in pediatric patients. Pediatric-specific thrombosis management guidelines should continue to evolve based on new research findings, with special emphasis on managing high-risk conditions, preventing long-term sequelae, and improving quality of life for affected children.

## 10. Conclusion

Pediatric thrombosis, though less common than in adults, presents a significant challenge due to its complex etiology, varied clinical manifestations, and the potential for long-term complications. The management of pediatric thrombosis requires a careful, individualized approach that accounts for the child’s unique physiology, underlying conditions, and age-related considerations. Early detection through vigilant screening, appropriate risk stratification, and prompt diagnosis are critical in preventing adverse outcomes.

## Author contributions

**Conceptualization:** Emmanuel Ifeanyi Obeagu.

**Methodology:** Emmanuel Ifeanyi Obeagu.

**Resources:** Emmanuel Ifeanyi Obeagu.

**Supervision:** Emmanuel Ifeanyi Obeagu.

**Validation:** Emmanuel Ifeanyi Obeagu.

**Visualization:** Emmanuel Ifeanyi Obeagu.

**Writing** – **original draft:** Emmanuel Ifeanyi Obeagu.

**Writing** – **review & editing:** Emmanuel Ifeanyi Obeagu.

## References

[1] Monagle P Cuello CA Augustine C. American Society of Hematology 2018 Guidelines for management of venous thromboembolism: treatment of pediatric venous thromboembolism. Blood Adv. 2018;2:3292–316. 30482766 10.1182/bloodadvances.2018024786 PMC6258911

[2] Manlhiot C Menjak IB Brandao LR. Risk, clinical features, and outcomes of thrombosis associated with pediatric cardiac surgery. Circulation. 2011;124:1511– 9. 21911785 10.1161/CIRCULATIONAHA.110.006304

[3] Giglia TM Massicotte MP Tweddell JS. Prevention and treatment of thrombosis in pediatric and congenital heart disease: a scientific statement from the American Heart Association. Circulation. 2013;128:2622–703. 24226806 10.1161/01.cir.0000436140.77832.7a

[4] Achey MA Nag UP Robinson VL. The developing balance of thrombosis and hemorrhage in pediatric surgery: clinical implications of age-related changes in hemostasis. Clin Appl Thromb Hemost. 2020;26:1076029620929092. 32584601 10.1177/1076029620929092 PMC7427005

[5] Spentzouris G Scriven RJ Lee TK Labropoulos N. Pediatric venous thromboembolism in relation to adults. J Vasc Surg. 2012;55:1785–93. 21944920 10.1016/j.jvs.2011.07.047

[6] Veldman A Nold MF Michel-Behnke I. Thrombosis in the critically ill neonate: incidence, diagnosis, and management. Vasc Health Risk Manag. 2008;4:1337–48. 19337547 10.2147/vhrm.s4274 PMC2663458

[7] George C Rahman M Monagle P. Challenges and opportunities in the pharmacological treatment of acute venous thromboembolism in children. Paediatr Drugs. 2020;22:385– 97. 32519267 10.1007/s40272-020-00403-5

[8] Monagle P. Diagnosis and management of deep venous thrombosis and pulmonary embolism in neonates and children. Semin Thromb Hemost. 2012;38:683–90. 23034828 10.1055/s-0032-1326784

[9] Eleftheriou D Ganesan V. Controversies in childhood arterial ischemic stroke and cerebral venous sinus thrombosis. Expert Rev Cardiovasc Ther. 2009;7:853–61. 19589121 10.1586/erc.09.41

[10] Günes AM Baytan B Günay U. The influence of risk factors in promoting thrombosis during childhood: the role of acquired factors. Pediatr Hematol Oncol. 2006;23:399–410. 16728360 10.1080/08880010600646324

[11] Alioglu B Avci Z Tokel K Atac FB Ozbek N. Thrombosis in children with cardiac pathology: analysis of acquired and inherited risk factors. Blood Coagul Fibrinolysis. 2008;19:294–304. 18469551 10.1097/MBC.0b013e3282fe73b1

[12] Barreirinho S Ferro A Santos M. Inherited and acquired risk factors and their combined effects in pediatric stroke. Pediatr Neurol. 2003;28:134–8. 12699865 10.1016/s0887-8994(02)00506-4

[13] Seligsohn U Lubetsky A. Genetic susceptibility to venous thrombosis. N Engl J Med. 2001;344:1222–31. 11309638 10.1056/NEJM200104193441607

[14] Souto JC Almasy L Borrell M. Genetic susceptibility to thrombosis and its relationship to physiological risk factors: the GAIT study. Am J Hum Genet. 2000;67:1452–9. 11038326 10.1086/316903 PMC1287922

[15] Ichiyama M Ohga S Ochiai M. Age-specific onset and distribution of the natural anticoagulant deficiency in pediatric thromboembolism. Pediatr Res. 2016;79:81– 6. 26372516 10.1038/pr.2015.180

[16] Toulon P Berruyer M Brionne-François M. Age dependency for coagulation parameters in paediatric populations. Thromb Haemost. 2016;116:9–16. 26988943 10.1160/TH15-12-0964

[17] Kumar R Sun LR Rodriguez V. Hemostatic and thrombotic considerations in the diagnosis and management of childhood arterial ischemic stroke: a narrative review. Semin Pediatr Neurol. 2022;43:101003. 36344025 10.1016/j.spen.2022.101003

[18] Chan A Lensing AW Kubitza D. Clinical presentation and therapeutic management of venous thrombosis in young children: a retrospective analysis. Thromb J. 2018;16:1–9. 30410424 10.1186/s12959-018-0182-4 PMC6211549

[19] Vieira JP Luis C Monteiro JP. Cerebral sinovenous thrombosis in children: clinical presentation and extension, localization and recanalization of thrombosis. Eur J Paediatr Neurol. 2010;14:80–5. 19201633 10.1016/j.ejpn.2008.12.004

[20] Knirsch W Kellenberger C Dittrich S. Femoral arterial thrombosis after cardiac catheterization in infancy: impact of Doppler ultrasound for diagnosis. Pediatr Cardiol. 2013;34:530–5. 22961345 10.1007/s00246-012-0488-0

[21] Lee EY Tse SK Zurakowski D. Children suspected of having pulmonary embolism: multidetector CT pulmonary angiography—thromboembolic risk factors and implications for appropriate use. Radiology. 2012;262:242–51. 22106353 10.1148/radiol.11111056

[22] Wagner MW Bosemani T Oshmyansky A Poretti A Huisman TA. Neuroimaging findings in pediatric cerebral sinovenous thrombosis. Childs Nerv Syst. 2015;31:705–12. 25715842 10.1007/s00381-015-2662-1

[23] Chan AK Deveber G Monagle P Brooker LA Massicotte PM. Venous thrombosis in children. J Thromb Haemost. 2003;1:1443–55. 12871279 10.1046/j.1538-7836.2003.00308.x

[24] Jaffray J Young G. Deep vein thrombosis in pediatric patients. Pediatr Blood Cancer. 2018;65:e26881. 10.1002/pbc.26881 29115714

[25] Goldenberg NA Knapp-Clevenger R Manco-Johnson MJ. Elevated plasma factor VIII and D-dimer levels as predictors of poor outcomes of thrombosis in children. N Engl J Med. 2004;351:1081–8. 15356305 10.1056/NEJMoa040161

[26] Nowak-Göttl U van Ommen H Kenet G. Thrombophilia testing in children: what and when should be tested? Thromb Res. 2018;164:75–8. 29518638 10.1016/j.thromres.2018.02.136

[27] Zia A Russell J Sarode R. Markers of coagulation activation, inflammation and fibrinolysis as predictors of poor outcomes after pediatric venous thromboembolism: a systematic review and meta-analysis. Thromb Res. 2017;160:1–8. 29078111 10.1016/j.thromres.2017.10.003 PMC5831248

[28] Chabrier S Husson B Dinomais M Landrieu P Tich SN. New insights (and new interrogations) in perinatal arterial ischemic stroke. Thromb Res. 2011;127:13–22. 21055794 10.1016/j.thromres.2010.10.003

[29] Lehman LL Rivkin MJ. Perinatal arterial ischemic stroke: presentation, risk factors, evaluation, and outcome. Pediatr Neurol. 2014;51:760–8. 25444092 10.1016/j.pediatrneurol.2014.07.031

[30] Fernández-López D Natarajan N Ashwal S Vexler ZS. Mechanisms of perinatal arterial ischemic stroke. J Cereb Blood Flow Metab. 2014;34:921–32. 24667913 10.1038/jcbfm.2014.41 PMC4050239

[31] Harteman JC Groenendaal F Kwee A Welsing PM Benders MJ de Vries LS. Risk factors for perinatal arterial ischaemic stroke in full-term infants: a case-control study. Arch Dis Child Fetal Neonatal Ed. 2012;97:F411–416. 22445901 10.1136/archdischild-2011-300973

[32] Bosenbark DD Krivitzky L Ichord R Jastrzab L Billinghurst L. Attention and executive functioning profiles in children following perinatal arterial ischemic stroke. Child Neuropsychol. 2018;24:106–23. 27599397 10.1080/09297049.2016.1225708

[33] Curry CJ Bhullar S Holmes J Delozier CD Roeder ER Hutchison HT. Risk factors for perinatal arterial stroke: a study of 60 mother-child pairs. Pediatr Neurol. 2007;37:99–107. 17675024 10.1016/j.pediatrneurol.2007.04.007

[34] Arnaez J Arca G Martín-Ancel A Agut T Garcia-Alix A. Neonatal arterial ischemic stroke: risk related to family history, maternal diseases, and genetic thrombophilia. Clin Appl Thromb Hemost. 2018;24:79–84. 29108421 10.1177/1076029617736383 PMC6714630

[35] Munoz D Hidalgo MJ Balut F. Risk factors for perinatal arterial ischemic stroke: a case–control study. Cell Med. 2018;10:2155179018785341. 32634191 10.1177/2155179018785341 PMC6172995

[36] Golomb MR MacGregor DL Domi T. Presumed pre‐or perinatal arterial ischemic stroke: risk factors and outcomes. Ann Neurol. 2001;50:163–8. 11506398 10.1002/ana.1078

[37] Darmency-Stamboul V Chantegret C Ferdynus C. Antenatal factors associated with perinatal arterial ischemic stroke. Stroke. 2012;43:2307–12. 22738921 10.1161/STROKEAHA.111.642181

[38] Sorg AL von Kries R Klemme M. Risk factors for perinatal arterial ischaemic stroke: a large case–control study. Dev Med Child Neurol. 2020;62:513–20. 31489622 10.1111/dmcn.14347

[39] Li C Miao JK Xu Y. Prenatal, perinatal and neonatal risk factors for perinatal arterial ischaemic stroke: a systematic review and meta‐analysis. Eur J Neurol. 2017;24:1006–15. 28646492 10.1111/ene.13337

[40] Srivastava R Dunbar M Shevell M. Development and validation of a prediction model for perinatal arterial ischemic stroke in term neonates. JAMA Netw Open. 2022;5:e2219203. 35767262 10.1001/jamanetworkopen.2022.19203 PMC9244611

[41] Martinez-Biarge M Cheong JL Diez-Sebastian J Mercuri E Dubowitz LM Cowan FM. Risk factors for neonatal arterial ischemic stroke: the importance of the intrapartum period. J Pediatr. 2016;173:62–8.e1. 27049002 10.1016/j.jpeds.2016.02.064

[42] Portale A Fiumara A Scalora L. Arterial ischemic stroke (AIS) in childhood: clinical report from a single control center. Childs Nerv Syst. 2019;35:283–93. 30542811 10.1007/s00381-018-4017-1

[43] Abdu H Seyoum G. Sex differences in stroke risk factors, clinical profiles, and in-hospital outcomes among stroke patients admitted to the medical ward of Dessie comprehensive specialized hospital, Northeast Ethiopia. Degener Neurol Neuromuscul Dis. 2022;12:133–44. 36304698 10.2147/DNND.S383564 PMC9595065

[44] Eng PC Tan LL Kimball TN Prapiadou S Tan BY. Ischemic stroke in women: understanding sex-specific risk factors, treatment considerations, and outcomes. J Cardiovasc Dev Dis. 2024;11:382. 39728272 10.3390/jcdd11120382 PMC11678625

[45] Govaert P Ramenghi L Taal R De Vries L DeVeber G. Diagnosis of perinatal stroke I: definitions, differential diagnosis and registration. Acta Paediatr. 2009;98:1556–67. 19663912 10.1111/j.1651-2227.2009.01461.x

[46] Lorenzano S Kremer C Pavlovic A. SiPP (Stroke in Pregnancy and Postpartum): a prospective, observational, international, multicentre study on pathophysiological mechanisms, clinical profile, management and outcome of cerebrovascular diseases in pregnant and postpartum women. Eur Stroke J. 2020;5:193–203. 32637653 10.1177/2396987319893512 PMC7313370

[47] Bogavac I Jeličić L Marisavljević M Bošković Matić T Subotić M. Arterial presumed perinatal ischemic stroke: a mini review and case report of cognitive and speech-language profiles in a 5-year-old girl. Children (Basel). 2023;11:33. 38255347 10.3390/children11010033 PMC10814911

[48] Bosenbark DD Krivitzky L Ichord R. Clinical predictors of attention and executive functioning outcomes in children after perinatal arterial ischemic stroke. Pediatr Neurol. 2017;69:79–86. 28274640 10.1016/j.pediatrneurol.2017.01.014

[49] Liu G Ding Q Wang B. Clinical and neuroimaging insights into childhood arterial ischemic stroke of different ages. Front Neurol. 2025;16:1568684. 40255892 10.3389/fneur.2025.1568684 PMC12005998

[50] Wu YW March WM Croen LA Grether JK Escobar GJ Newman TB. Perinatal stroke in children with motor impairment: a population-based study. Pediatrics. 2004;114:612–9. 15342829 10.1542/peds.2004-0385

[51] Reiter PD Wathen B Valuck RJ Dobyns EL. Thrombosis risk factor assessment and implications for prevention in critically ill children. Pediatr Crit Care Med. 2012;13:381– 6. 22198812 10.1097/PCC.0b013e31823893f5

[52] Abdelghani E Cua CL Giver J Rodriguez V. Thrombosis prevention and anticoagulation management in the pediatric patient with congenital heart disease. Cardiol Ther. 2021;10:325–48. 34184214 10.1007/s40119-021-00228-4 PMC8555036

[53] Branchford BR Betensky M Goldenberg NA. Pediatric issues in thrombosis and hemostasis: the how and why of venous thromboembolism risk stratification in hospitalized children. Thromb Res. 2018;172:190–3. 29472108 10.1016/j.thromres.2018.02.010

[54] Hanison E Corbett K. Non-pharmacological interventions for the prevention of venous thromboembolism: a literature review. Nurs Stand. 2016;31:48–57. 10.7748/ns.2016.e10473 27808636

[55] Hennerici MG Kern R Szabo K. Non-pharmacological strategies for the treatment of acute ischaemic stroke. Lancet Neurol. 2013;12:572–84. 23684083 10.1016/S1474-4422(13)70091-7

[56] Putri SZ Budu B Gemini G Natsir R. Non-pharmacological interventions for anemia treatment: systematic review. Muhammadiyah Int Public Health Med Proc. 2021;1:283–312.

[57] Betensky M Bittles MA Colombani P Goldenberg NA. How we manage pediatric deep venous thrombosis. Semin Intervent Radiol. 2017;34:35–49. 28265128 10.1055/s-0036-1597762 PMC5334487

[58] Radulescu VC. Management of venous thrombosis in the pediatric patient. Pediatric Health Med Ther. 2015;111:111–9. 10.2147/PHMT.S65697 PMC5683259 29388593

[59] McGrady ME Todd K Ignjatovic V. Results of an international survey on adherence with anticoagulation in children, adolescents, and young adults: communication from the ISTH SSC Subcommittee on Pediatric and Neonatal Thrombosis and Hemostasis. J Thromb Haemost. 2022;20:1720–8. 35427434 10.1111/jth.15730

[60] Athale U. Thrombosis in pediatric cancer: identifying the risk factors to improve care. Expert Rev Hematol. 2013;6:599–609. 24125523 10.1586/17474086.2013.842124

[61] Levy-Mendelovich S Cohen O Klang E Kenet G. 50 years of pediatric hemostasis: knowledge, diagnosis, and treatment. Semin Thromb Hemost. 2023;49:217–24. 36174607 10.1055/s-0042-1756704

[62] deVeber G Chan A Monagle P. Anticoagulation therapy in pediatric patients with sinovenous thrombosis: a cohort study. Arch Neurol. 1998;55:1533–7. 9865797 10.1001/archneur.55.12.1533

[63] Andrew M Marzinotto V Brooker LA. Oral anticoagulation therapy in pediatric patients: a prospective study. Thromb Haemost. 1994;71:265–9. 8029786

[64] Dabbous MK Sakr FR Malaeb DN. Anticoagulant therapy in pediatrics. J Basic Clin Pharm. 2014;5:27–33. 25031496 10.4103/0976-0105.134947 PMC4074692

[65] Monagle P Newall F. Management of thrombosis in children and neonates: practical use of anticoagulants in children. Hematology Am Soc Hematol Educ Program. 2018;2018:399–404. 30504338 10.1182/asheducation-2018.1.399 PMC6245972

[66] Bulut Y Sapru A Roach GD. Hemostatic balance in pediatric acute liver failure: epidemiology of bleeding and thrombosis, physiology, and current strategies. Front Pediatr. 2020;8:618119. 33425821 10.3389/fped.2020.618119 PMC7786276

[67] Eckman MH Erban JK Singh SK Kao GS. Screening for the risk for bleeding or thrombosis. Ann Intern Med 2003;138:W15–24. 12558384 10.7326/0003-4819-138-3-200302040-00011-w1

[68] Lassandro G Palmieri VV Palladino V Amoruso A Faienza MF Giordano P. Venous thromboembolism in children: from diagnosis to management. Int J Environ Res Public Health. 2020;17:4993. 32664502 10.3390/ijerph17144993 PMC7400059

[69] Streif W Andrew ME. Venous thromboembolic events in pediatric patients: diagnosis and management. Hematol Oncol Clin North Am. 1998;12:1283–312, vii. 9922936 10.1016/s0889-8588(05)70053-8

[70] Bauman ME Massicotte MP Ray L Newburn-Cook C. Developing educational materials to facilitate adherence: pediatric thrombosis as a case illustration. J Pediatr Health Care. 2007;21:198–206. 17478312 10.1016/j.pedhc.2007.02.011

[71] Obeagu EI Obeagu GU. Thromboinflammation in COVID-19: unraveling the interplay of coagulation and inflammation. Medicine (Baltim). 2024;103:e38922. 10.1097/MD.0000000000038922 PMC11245273 38996158

[72] Obeagu EI Obeagu GU Aja PM. Soluble platelet selectin and platelets in COVID-19: a multifaceted connection. Ann Med Surg (Lond). 2024;86:4634–42. 39118706 10.1097/MS9.0000000000002302 PMC11305715

[73] Obeagu EI Tukur M Akaba K. Impacts of COVID-19 on hemostasis: coagulation abnormalities and management perspectives. Ann Med Surg (Lond). 2024;86:5844–50. 39359765 10.1097/MS9.0000000000002237 PMC11444586

